# Chemical Profile and Bioinsecticidal Nanoemulsion of *Haplopappus foliosus* Essential Oil: Mechanistic Insights into Pest Management Using a *Drosophila* Model

**DOI:** 10.3390/plants15091282

**Published:** 2026-04-22

**Authors:** Valentina Silva, Evelyn Muñoz, Susana Flores, Constanza Reyes, Natalie Bravo, Héctor A. Levipan, Iván Montenegro, Julio Alarcón, Rocío Santander, Alejandro Madrid

**Affiliations:** 1 Laboratorio de Productos Naturales y Síntesis Orgánica (LPNSO), Facultad de Ciencias Naturales y Exactas, Universidad de Playa Ancha, Subida Leopoldo Carvallo 270, Valparaíso 2340000, Chile; silvapedrerosv@gmail.com (V.S.); s.flores.gonzalez@gmail.com (S.F.); reyesveraconstanza@gmail.com (C.R.); natalie.bravo@postgrado.uv.cl (N.B.); 2 Departamento de Química Orgánica, Facultad de Ciencias Químicas, Universidad de Concepción, Concepción 4070371, Chile; emunoznu@udec.cl; 3 Laboratorio de Ecopatología y Nanobiomateriales, Departamento de Ciencias y Geografía, Facultad de Ciencias Naturales y Exactas, Universidad de Playa Ancha, Subida Leopoldo Carvallo 270, Valparaíso 2340000, Chile; hector.levipan@upla.cl; 4 Center of Interdisciplinary Biomedical and Engineering Research for Health (MEDING), Escuela de Obstetricia y Puericultura, Facultad de Medicina, Universidad de Valparaíso, Angamos 655, Viña del Mar 2520000, Chile; ivan.montenegro@uv.cl; 5 Grupo de Investigación Química y Biotecnología de Productos Naturales Bioactivos, Laboratorio de Síntesis y Biotransformación de Productos Naturales, Departamento de Ciencias Básicas, Facultad de Ciencias, Universidad del Bío-Bío, Chillán 3800708, Chile; jualarcon@ubiobio.cl; 6 Laboratorio de Cinética y Fotoquímica, Departamento de Ciencias del Ambiente, Facultad de Química y Biología, Universidad de Santiago de Chile, Santiago 9160000, Chile; rocio.santanderm@usach.cl

**Keywords:** Bailahuén, essential oil, acetylcholinesterase, larvicidal, *Drosophila melanogaster*, molecular docking

## Abstract

The increasing demand for sustainable pest management has positioned essential oils (EOs) as viable bio-based alternatives to synthetic pesticides. This study investigates the insecticidal potential of *Haplopappus foliosus* EO, a Chilean endemic medicinal plant, against *Drosophila melanogaster* as a key toxicological model for fruit fly control. Chemical characterization identified 56 compounds, with 4-terpineol (27.27%) and α-bisabolol (10.40%) as the primary constituents, marking the first report of α-bisabolol in this species. To enhance bioavailability and overcome EO volatility, a nanoemulsion was developed, achieving an exceptionally small and stable particle size of 2.10 nm that remained consistent for over 90 days. Nanoencapsulation significantly optimized the EO’s efficacy, reducing the median lethal concentration (LC_50_) from 120.26 µg/mL to a potent 54.57 µg/mL. While in vitro assays showed the free oil as a more potent acetylcholinesterase (AChE) inhibitor, molecular docking confirmed the high affinity of 4-terpineol and α-bisabolol for the enzyme’s active site, elucidating the neurotoxic mechanism at a molecular level. In silico analysis predicted a favorable human safety profile within GHS classes 4 and 5. Overall, this stable nanoformulation represents a sustainable biotechnological strategy for agricultural pest management, leveraging the synergistic effects and enhanced delivery of natural products.

## 1. Introduction

*Drosophila* species are significant pests impacting both domestic and industrial sectors [1]. *Drosophila melanogaster*, an African-originated species also known as the fruit fly or vinegar fly, is globally recognized as a cost-effective model organism; beyond its foundational role in genetics, it serves as a critical model for studying toxicology as well as infectious and neurodegenerative diseases [1,2]. In agricultural contexts, *D. melanogaster* primarily affects damaged or decaying fruit; furthermore, this species acts as a key vector for sour rot in grapevines. Adults transmit the “*Drosophila* complex”, a pathogenic consortium including *Kloeckera apiculata*, *Saccharomycopsis vini*, and *Acetobacter* spp., among others. The adults lay eggs on fruit pulp and the larvae prevent the healing of wounds due to their movement, which favors the penetration of microorganisms [3].

Similarly, species such as *D. suzukii* cause devastating agricultural damage due to their ability to oviposit on healthy fruit using a serrated ovipositor. This morphological feature differentiates it from *D. melanogaster* and allows it to bore into undamaged fruit [4], making it a major threat to the industry. Global data from the USA, Italy, Brazil, Chile, and Switzerland indicate that *Drosophila*-related losses account for 20% of affected crops and the financial burden is particularly severe for berries and cherries, with estimated damages ranging from $421.5 to $511.3 million USD for these crops alone [5]. To mitigate these impacts, chemical control has been systematically utilized as the primary strategy, employing active ingredients such as acetamiprid and thiacloprid, as well as organophosphates (malathion, phosmet) and pyrethroids (zeta-cypermethrin, bifenthrin) [6]. However, many of these treatments have become a problem for the export market, as harvested fruit may exceed the maximum residue limits (MRLs) established by importing countries [7]. This issue, coupled with the emergence of resistance mechanisms and adverse effects on non-target species, emphasizes the urgent search for novel control strategies [8].

Among the various alternatives, EOs are a promising option, having shown efficacy across different developmental stages of *Drosophila* spp. [9,10,11]. The chemical diversity of EOs provides multiple mechanisms of action; however, the predominant pathways involve the inhibition of either AChE or gamma-aminobutyric acid (GABA) receptors [10]. Oregano and thyme EOs stand out for their insecticidal activity, particularly due to the presence of carvacrol, a naturally occurring terpene that exhibits toxicity to insects through the GABAergic system, as well as through the inhibition of AChE, but also via glutathione-S-transferase, carboxylesterase, α-amylase, and lipase, among other mechanisms [12,13,14].

Notably, AChE is a highly conserved molecular target across the *Drosophila* genus, exhibiting high sequence homology between *D. melanogaster* and *D. suzukii*, which justifies the use of the former as a reliable toxicological model [15]. Bioinsecticides derived from EOs are promising alternatives due to their natural origin and biodegradability, minimizing environmental persistence. Furthermore, their lipophilic nature facilitates permeation through insect membranes, potentially enhancing efficacy [10]. To address challenges regarding low stability and high volatility of EOs, nanoemulsions have been proposed. Nanoencapsulation can optimize pest control by providing a controlled release of bioactive components, boosting biological activity and extending shelf life [16].

The genus *Haplopappus* comprises a diverse group of South American species that are widely recognized in ethnomedicine as “Bailahuén”. Several species in this genus possess a distinctive resinous coating that acts as a defensive antimicrobial barrier and a physical adhesive trap against pests [17,18]. *Haplopappus foliosus*, a Chilean endemic medicinal shrub, has demonstrated significant insecticidal potential; for instance, its EO has been evaluated as a botanical fumigant against *Musca domestica*, exhibiting a median lethal concentration of 4.43 mg/dm^3^ of air [19]. This potent bioactivity highlights the potential of *H. foliosus* for developing sustainable pest management strategies. Despite these promising attributes, the development of nanoscale delivery systems for *H. foliosus* metabolites and their specific interactions with the *Drosophila* cholinergic system remain unexplored.

Given the need for sustainable alternatives and the phytochemical potential of *H. foliosus*, this study focused on evaluating its effect on *D. melanogaster*, which is a versatile model for insecticide toxicology [20]. The main objective of this research was to evaluate the insecticidal activity of *H. foliosus* EO, its nanoemulsion and its major components against *D. melanogaster* larvae. Additionally, the inhibitory capacity of these treatments on AChE was determined, integrating in silico molecular docking analysis to elucidate the interactions at the molecular level and provide insights into their potential application in postharvest protection.

## 2. Results

### 2.1. EO Composition

*Haplopappus foliosus* EO obtained via Clevenger extraction was pale yellow, with a yield of 0.04% (*w*/*w*). A total of 56 compounds were identified by GC-MS, representing 98.05% of the oil. The composition of the EO, shown in Table 1, reveals that the chemical profile of the *H. foliosus* EO is characterized by a high proportion of oxygenated terpenoids, which accounted for 75.86% of the total composition. These were distributed into oxygenated monoterpenes (36.17%) and oxygenated sesquiterpenes (39.69%); hydrocarbon sesquiterpenes followed as the third most abundant group, representing 11.64% of the oil.

The major constituents were 4-terpineol (27.27%) and α-bisabolol (10.40%), other significant components included spathulenol (6.70%), α-eudesmol (4.95%), and muurola-4,10(14)-dien-1β-ol (3.77%). Additionally, the EO contained a fraction of phenylpropanoids (2.45%) represented by methyleugenol (1.33%) and eugenol (1.12%), as well as minor amounts of hydrocarbon monoterpenes (3.36%), monoterpene esters (1.24%), fatty aldehydes (1.48%), hydrocarbon alkanes (0.94%), phenols (0.58%), fatty alcohols (0.27%), and fatty acids (0.23%).

### 2.2. Nanoemulsion Characterization

The parameters presented in Table 2 are relevant for evaluating the stability of the *H. foliosus* nanoemulsion. The findings offer insights into the nanoemulsion capacity to preserve its physicochemical properties over time, a crucial consideration when assessing its potential application in the agricultural sector, particularly in view of the fact that it will be subjected to storage, transportation, and field application processes.

The EO nanoformulation maintained a near-neutral pH and achieved an exceptionally small particle size of 2.10 nm, well within the range required for nanoemulsion classification [21]. Notably, this loaded formulation exhibited a smaller particle size than the unloaded (blank) control, which recorded a size of 2.80 nm. However, it is important to clarify that while emulsions with sizes smaller than 20 nm, such as in this case, could be classified as microemulsions, the two terms should not be used as synonyms. Unlike nanoemulsions, microemulsions are thermodynamically stable colloidal dispersions and, hence, form spontaneously upon mixing of components. Nanoemulsions, by contrast, are kinetically stable systems that typically require external energy input for their formation [22].

Regarding the heterogeneity of the formulation, the polydispersity index (PDI) of both samples was close to 0.200, which proves the homogeneity of the system [23]. A sharp, narrow intensity peak was observed in the size distribution analysis, indicating a low PDI and confirming the monodisperse nature of the nanodroplets. Regarding the surface charge, both samples exhibited negative zeta potentials (ZP) of −3.38 ± 0.2 mV and −3.44 ± 0.1 mV for the loaded and unloaded nanoemulsions, respectively, being close to neutrality.

To assess stability after storage, the empty and loaded nanoformulations were kept for 90 days at room temperature and a second DLS characterization was performed; additionally, phase separation was analyzed under different storage conditions. These results are presented in Table 3.

The prepared formulations were examined for emulsion stability before storage; physical stability assessments revealed that the formulations maintained their integrity under both low-temperature and heat-tropical storage. No instability phenomena, such as coalescence or creaming, were observed after temperature cycling tests, indicating that the nanoemulsion is stable and resistant to environmental fluctuations. The findings of the new characterization demonstrate that the *H. foliosus* EO-loaded nanoemulsion maintained its configuration, since it retained an optimum PDI of 0.200 and a particle size that remained comparable to the values recorded seven days after formulation, a feature that was not replicated by the empty formulation. Following a period of 90 days storage, a marked increase in particle size was observed in the empty nanoemulsion. This finding suggests that the constituents of the EO play a crucial role in stabilizing the system, overall, the preservation of the nanometric size and monodispersity after 90 days of shelf-life underscores the potential of this formulation as a durable and reliable candidate for botanical insecticide development.

### 2.3. Insecticidal Potential

The larvicidal activity of *H. foliosus* EO, its nanoemulsion and main compounds are presented in Table 4. The results show that all samples were active on *D. melanogaster* larvae, with the exception of the unloaded nanoemulsion, confirming that the insecticidal activity is attributed to the encapsulated EO and not to the components of the nanoformulation matrix.

Although the *H. foliosus* EO presented a similar activity to the positive control carvacrol (119.91 µg/mL), better results were obtained due to its encapsulation. The nanoemulsion of the *H. foliosus* EO reduced the concentration necessary to achieve 50% mortality of the larvae, reducing its LC_50_ from 120.26 µg/mL to 54.57 µg/mL. It is also relevant to note that the nanoemulsion outperformed the isolated compounds 4-terpineol (76.85 µg/mL) and α-bisabolol (82.36 µg/mL) in efficacy.

The results of the evaluation of the EO, its nanoemulsion, and major compounds regarding the inhibition of the AChE enzyme are summarized in Table 5.

Analysis of variance (ANOVA) showed highly significant differences in values among the evaluated treatments (*p* < 0.005). In contrast to the observations made in larvicidal assays, the nanoencapsulation of the EO did not result in a potentiation of the inhibitory activity on the AChE enzyme. The free *H. foliosus* EO exhibited a lower IC_50_ (115.39 ± 2.2 µg/mL) compared to the loaded nanoemulsion (166.85 ± 1.6 µg/mL). The empty nanoemulsion exhibited no inhibitory activity, thereby confirming that the formulation does not induce inhibitory effect. Carvacrol, used as a positive control, presented the highest inhibitory activity with an IC_50_ of 55.93 ± 0.6 µg/mL, followed closely by the isolated compounds 4-terpineol (77.13 ± 1.3 µg/mL) and α-bisabolol (89.76 ± 0.5 µg/mL).

### 2.4. In Silico Prediction of Physicochemical Properties, ADME, and Toxicity

The physicochemical properties and ADME-related parameters of the major evaluated compounds were predicted using the SwissADME platform and are summarized in Table 6. All analyzed compounds exhibited moderate lipophilicity values (LogP ranging from 2.6 to 3.8), suggesting an adequate capacity for permeation across biological membranes. In addition, all compounds showed low topological polar surface area values (TPSA = 20.23 Å^2^), together with a limited number of hydrogen bond donors and acceptors (HBD = 1; HBA = 1), features that are consistent with the high gastrointestinal absorption predicted for all cases [24].

Regarding molecular flexibility, the compounds presented between 1 and 4 rotatable bonds, with α-bisabolol being the most flexible molecule. Aqueous solubility was classified as poor for all evaluated compounds, in agreement with their lipophilicity values. Furthermore, none of the compounds displayed PAINS alerts, indicating the absence of substructures commonly associated with nonspecific interference in biological assays [25,26].

The results of the toxicological predictions obtained using ProTox-III are presented in Table 7. The estimated oral toxicity values indicated that all compounds fall within low acute toxicity categories, with values (LD_50_) ranging from 810 mg/kg for carvacrol to 2830 mg/kg for α-bisabolol.

In terms of specific toxicological endpoints, most compounds were predicted to be inactive with respect to hepatotoxicity, neurotoxicity, nephrotoxicity, and cardiotoxicity. However, the positive control carvacrol was predicted to be active for neurotoxicity and nephrotoxicity. Finally, all evaluated compounds were predicted to be capable of crossing the blood–brain barrier, which is consistent with their physicochemical profiles, particularly their low TPSA values and moderate lipophilicity [27,28,29].

### 2.5. Molecular Docking

The molecular docking results of the major compounds present in the EO samples of *H. foliosus* against AChE from *D. melanogaster* are summarized in Table 8. The predicted binding free energy (ΔG) values revealed clear differences in ligand affinity toward the enzyme.

**Figure 1:**
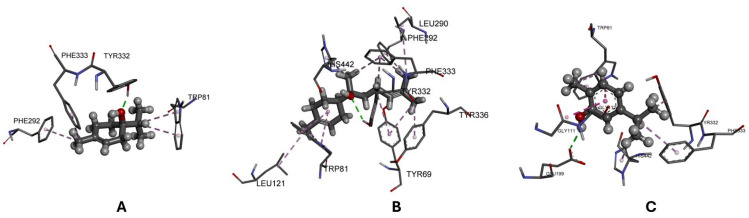
Three-dimensional representations of the molecular docking interactions between the major compounds and carvacrol within the active site of *Drosophila melanogaster* acetylcholinesterase (Dm-AChE). (**A**) Dm-AChE-4-terpineol complex; (**B**) Dm-AChE-α-bisabolol complex; (**C**) Dm-AChE-carvacrol complex. The amino acid residues involved in ligand recognition are shown as gray sticks and are labeled accordingly. Ligands are represented as ball-and-stick models with carbon atoms in gray, oxygen atoms in red, and hydrogen atoms in light gray for clarity. Hydrogen bonds are indicated with green dashed lines, hydrophobic alkyl interactions with pink dashed lines, and π-π interactions with purple dashed lines.

Among the evaluated compounds, 4-terpineol exhibited the highest affinity, with a binding energy of −8.6 kcal/mol, followed by α-bisabolol (−8.0 kcal/mol). In contrast, carvacrol showed less favorable binding energies, with values of −5.7 and −5.0 kcal/mol, respectively. A detailed analysis of the protein-ligand interactions allowed the identification of key contacts responsible for the stabilization of the formed complexes (Figure 1).

In the case of carvacrol, the binding mode was characterized by the formation of a hydrogen bond with Glu199 at 2.14 Å, accompanied by π-π stacking interactions with Trp81 and Gly111, as well as hydrophobic alkyl contacts with Tyr332, His442, and Phe333. Overall, these results indicate that the differences in predicted binding affinity among the compounds are associated with the nature and extent of the polar and hydrophobic interactions established within the acetylcholinesterase active site, particularly those involving key aromatic residues.

## 3. Discussion

The chemical composition of *H. foliosus* EO has been previously documented, yet our findings reveal significant qualitative and quantitative variations compared to the report by Urzúa et al. (2010) [19]. While the authors have identified limonene (28.0%) and epi-bicyclosesquiphellandrene (9.84%) as the primary constituents [19], these compounds were absent in our EO. Furthermore, although 4-terpineol was identified in both studies, it emerged as the major component in our oil (27.27%), contrasting with the 6.36% reported previously. The differences in the relative presence of different metabolites may be due to environmental factors such as the pressures of the xerophytic coastal biome. In the same context, Villagra et al. (2021) analyzed the differences in the secondary metabolites of *H. foliosus* when its branches were affected by gall-induced insects [30]. The authors demonstrated that the composition changes when the plant is parasitized, with an increased proportion of monoterpenes such as limonene. The expression of other monoterpene like *p*-cymene, α-thujene and γ-terpinene and the oxygenated monoterpene 4-terpineol was mostly found in the healthy apical branches of *H. foliosus*. The aforementioned studies do not report the presence of α-bisabolol, nor has it been reported in research on extracts or resinous exudates of *H. foliosus* [31,32,33,34,35]. Therefore, this would be the first description of the presence of α-bisabolol in *H. foliosus*, which is important considering its widespread use as a dermatological ingredient due to its anti-inflammatory, anti-irritant, antibacterial and non-allergenic properties [36]. Standardizing the chemical profile of the EO remains a challenge due to environmental variability. Further research is required to map the chemotype distribution of *H. foliosus* across seasonal cycles in order to ensure a standardized supply for replication.

According to the results shown in Table 2, the *H. foliosus* nanoemulsion achieved an optimal particle size, as these typically have a maximum size of 200 nm [21]. However, a value of 2.10 nm is exceptionally small when compared to other EO nanoemulsions reported in the literature. For instance, studies on *Citrus medica* reached minimum droplet sizes of 73 nm [37], while other formulations using non-ionic surfactants like Tween 80 or Span 80 reported average sizes of 222 nm for sage oil and 32 nm for *Syzygium aromaticum* [38,39]. Sizes approaching the 10 nm range have typically been reserved for isolated compounds [40] or formulations characterized by the use of co-stabilizers such as saponins [41]. In some authors’ definitions, a particle size of 2.10 nm, as achieved by the *H. foliosus* nanoemulsion, would fall into the microemulsion category. However, it is a term that has become unpopular because particle size alone is not a reliable criterion for discriminating between micro and nanoemulsions and has been applied with little rigor [42,43], since, as mentioned, microemulsions are thermodynamically stable formulations that form spontaneously.

This exceptional particle size not only positions the formulation within nanotechnology standards but is also complemented by a highly controlled size distribution. The PDI serves as a critical indicator of formulation uniformity, a PDI below 0.300 typically suggests a narrow and stable distribution of the oil within the aqueous phase [23]. The *H. foliosus* nanoemulsion exhibited a PDI of 0.200, reflecting a highly homogeneous system. This is significant because, in excessively heterogeneous systems, droplet aggregation often leads to physical instability, such as creaming or phase separation. Furthermore, the inclusion of medium- or long-chain triglycerides, such as the olive oil used in this formulation, enhances stability by mitigating Ostwald ripening which is recognized as the primary instability mechanism in EO nanoemulsions [44,45].

While these structural components ensure physical integrity, the surface electronic properties of the droplets also play a defining role in the system’s behavior. The electrical properties of a nanoemulsion are usually characterized by the ZP, which varies from −60 to +60 mV depending on the emulsifier’s nature; higher values of the zeta potential contribute to greater stability, as they prevent flocculation [45]. Although the zeta potential of the *H. foliosus* nanoemulsion does not reach ±30 mV value (used to indicate moderate stability of the colloidal system) and is closer to neutrality [46], no flocculation phenomena were observed. This suggests that the system stability is primarily governed by steric stabilization provided by the used non-ionic surfactants rather than electrostatic repulsion [47].

In larvicidal assays, nanoencapsulation yielded significant improvements in insecticidal activity, this superiority suggests a synergistic effect between the constituents of the EO that is enhanced by the release system, which protects them from degradation, thus optimizing their bioavailability of the active components. Such increased efficacy is likely driven by the small particle size, which facilitates superior cuticle penetration in the larvae and ensures a more uniform distribution within the culture medium [48]. In this context, the nanoemulsion serves as a protective matrix for volatile bioactive agents, such as 4-terpineol, preventing premature degradation and maximizing their insecticidal effect.

The biological activity observed for this compound is consistent with findings by Huang et al. (2022), who also demonstrated that 4-terpineol exerts a multi-targeted mechanism of action [49]. This molecule effectively modulates several key enzymes, notably inhibiting AChE and GST, while also altering catalase and Na+/K+-ATPase levels [49,50]. Furthermore, recent research has indicated that 4-terpineol can disrupt biological processes associated with oxidative stress [51], broadening its profile as a potent biological control agent. While 4-terpineol is a promising candidate for pest management due to its low environmental persistence and minimal residual hazard [52], its inherent volatility remains a challenge. Consequently, the nanoencapsulation of the 4-terpineol-rich oil presented in this study offers an effective strategy to overcome these limitations, maximizing its insecticidal potential while minimizing the need for excessive applications.

For α-bisabolol, previous research has addressed the insecticidal activity of EOs with a high content of this sesquiterpene, like *Vanillosmopsis pohlii* EO, demonstrating efficacy against fruit pests such as whiteflies (*Bemisia argentifolii*) [53]. The larvicidal effect of this molecule against *Aedes aegypti* has been the subject of evaluation [54]; however, the bioactivity of the substance is typically enhanced when it is part of an EO rather than when it acts alone [44]. Moreover, studies on α-bisabolol have classified it as a penetration enhancer in transdermal delivery and it has been categorized as a safe substance to be used in formulations [36,55]; however, its low water solubility significantly limits its pharmaceutical and biotechnological applications [56], a challenge that nanoformulation seeks to address. Additionally, it can be inferred that other metabolites present in the oil, such as carvacrol, linalool, *p*-cymene, eugenol, and caryophyllene, would enhance the overall insecticidal action of the extract. Although these constituents are present in minor proportions, they act synergistically by modulating multiple neurotoxic targets, primarily through the inhibition of the AChE enzyme and the facilitation of cuticular penetration in the insect—phenomena extensively documented in the literature for complex terpenoid mixtures [57,58,59].

The multiple mechanisms of action are fundamental from an agronomic perspective, as the use of natural mixtures could significantly delay the development of resistance in *D. melanogaster* populations compared to conventional single-component treatments, which exert acute selective pressure on a single biological target. In contrast, when evaluating efficacy against established standards, malathion (M+) exhibited the highest insecticidal activity (LC_50_ = 0.11 µg/mL). This difference in potency—approximately three orders of magnitude relative to the crude essential oil—is consistent with the nature of malathion as a highly specialized organophosphate designed for the irreversible inhibition of AChE [60]. Thus, while the *H. foliosus* EO offers a strategic advantage due to its complexity and lower risk of resistance, the synthetic standard represents the upper limit of optimized biological potency.

Following the demonstration of the insecticidal efficacy of *H. foliosus* EO, its nanoemulsion, and its primary constituents, we sought to elucidate the underlying mechanism of action. Inhibition of the enzyme AChE was selected as the target of study, due to its critical role in nerve impulse transmission in the central nervous system of insects. The inhibition of AChE leads to the synaptic accumulation of acetylcholine, resulting in cholinergic overstimulation, paralysis, and ultimately death. As conventional insecticides, such as organophosphates and carbamates, target this enzyme [61], this assay provides insight into whether the observed activity is mediated by cholinergic dysfunction.

The results obtained in the AChE inhibition assays shown in Table 5 confirm the activity of carvacrol as a positive control (55.93 ± 0.6 µg/mL), which is consistent with previous literature. For instance, Jukić et al. (2007) determined the median inhibitory concentration of carvacrol to be 63 µg/mL [62], whereas Muñoz (2024) reported it to be 40 µg/mL [63]. Carvacrol stands out as a potent control agent for various pests due to its ability to inhibit GST activity, suppress cytochrome P450 enzymes, and interfere with microRNA and AChE activity [64]. Furthermore, 4-terpineol exhibited an IC_50_ of 77.13 ± 1.3 µg/mL. This molecule ability to inhibit the AChE enzyme has been documented in other biological models, including adults of the rice weevil [65] and *Plutella xylostella*, the latter showing a significant decrease in enzymatic activity following fumigation with this terpene [49]. Additionally, α-bisabolol contributes to the overall anticholinesterase effect and has been shown to reversibly inhibit the α-nicotinic acetylcholine receptor. Notably, increasing acetylcholine concentrations does not reverse this inhibition, suggesting a non-competitive mechanism [66].

The EO proved to be more effective than the nanoemulsion at inhibiting AChE, unlike its performance against larvae. This discrepancy between efficacy in an in vivo assay versus enzymatic assays has been previously reported in the literature. Studies with EO nanoemulsions of basil, cumin, marjoram and chamomile showed enhanced toxicity on the insect *Aphis craccivora*; however, this effect was not replicated in enzymatic assays, where the free EOs showed greater activity [67]. A similar phenomenon has been observed with *Mentha spicata* EO and its major constituent, carvone (81.8%); while its nanoemulsion exhibits superior potency in aphid bioassays, its AChE inhibitory activity is notably lower than that of the free compound [68]. These findings lead us to believe that although the nanoemulsion optimizes the delivery and bioavailability of the active ingredient in the larvae, under in vitro enzymatic assay conditions, the encapsulation may restrict contact with the active site of the enzyme, where ironically, the protection afforded by the nanoemulsion limits the immediate availability of the oil. Although the in vitro enzymatic assays showed lower AChE inhibition for the nanoformulation, these results must be interpreted within the complex context of in vivo larval toxicity. It is important to state that AChE inhibition is likely one of several contributing mechanisms to larval mortality. The superior performance of the nanoemulsion in larvae suggests a synergistic effect driven by enhanced delivery and bioavailability [16]. Furthermore, under in vivo conditions, larvae are exposed to the formulation through multiple pathways, including ingestion and direct cuticular contact, which are not captured in static enzymatic assays. The nanoemulsion matrix also plays a critical role in the release kinetics by preventing the premature volatilization and degradation of volatile bioactive agents such as 4-terpineol and the formulation maximizes its long-term insecticidal efficacy.

In silico tests on the physicochemical properties of the constituents of *H. foliosus* EO (Table 6) confirm its low solubility in water. This characteristic represents a critical technological challenge for the application of EOs as insecticides, since the active principles must be efficiently dispersed or solubilized in aqueous media for their use in the field. For a botanically derived product to be commercially viable in agriculture, it is imperative that it overcomes this limitation and maintains its structural integrity without degrading during storage. In this context, the developed nanoemulsion has been shown to overcome this barrier, exhibiting superior physical and chemical stability, as detailed in the results of Table 2 and Table 3. On the other hand, the predictive toxicological analysis (Table 7) reveals a significant comparative advantage, as the estimated toxicity of the positive control carvacrol was higher than that predicted for the major components of *H. foliosus*. In this context, the in silico predictions suggest a favorable preliminary human safety profile, with the major compounds falling within GHS toxicity classes 4 and 5. These results indicate a potentially lower acute toxicity risk for applicators and consumers compared with carvacrol. However, these findings should be interpreted as an initial computational approximation and do not replace dedicated experimental toxicological and ecotoxicological assessments, particularly with respect to non-target organisms. Overall, these results suggest that the analyzed compounds exhibit favorable in silico ADME profiles and generally low predicted toxicity, although compound-specific differences may be relevant for subsequent biological evaluations.

The molecular docking studies conducted and shown in Table 8 indicated that 4-terpineol and α-bisabolol exhibit the most favorable binding affinities toward *Dm*-AChE, stabilizing the complexes primarily through a combination of hydrogen bonds and hydrophobic interactions with conserved aromatic residues within the active site. This interaction pattern is consistent with recent reports describing the ability of oxygenated monoterpenes and sesquiterpenes to interact effectively with AChE and modulate its biological activity [69]. Likewise, the recurrence of key residues in the formed complexes agrees with current studies highlighting their relevance in the stabilization of inhibitors of this enzyme [70], whereas the lower affinities observed for carvacrol may be attributed to a less favorable structural fit within the catalytic pocket, as described for other terpenoid compounds [71]. Nevertheless, as molecular docking constitutes a theoretical and essentially static approach that does not explicitly account for dynamic, entropic, or biological environment effects, the obtained results should be interpreted as exploratory evidence aimed at rationalizing affinity trends rather than as a definitive quantitative measure of biological activity.

Having gathered all this new evidence, we are inclined to suggest that while *D. melanogaster* serves as a valid toxicological model, these findings have significant implications for managing economically relevant pests such as *D. suzukii*. The high degree of conservation in AChE sequences and neurophysiological pathways within the *Drosophila* genus [15] suggests that the bioinsecticidal effects observed here could be translated to target species that affect global fruit production. However, further validation tests using bioassays involving *D. suzukii* or other agricultural pests, in which the *H. foliosus* nanoformulation is sprayed onto crops, are needed to verify its effectiveness in environments that simulate field conditions, including phytotoxicity studies to ensure crop safety, and toxicity studies to confirm the safety of the EO for humans and other non-target organisms.

## 4. Materials and Methods

### 4.1. Extraction of EO

The aerial parts of *H. foliosus* were collected from wild populations in coastal Mediterranean sclerophyllous scrubland in Pichidangui, Los Vilos, Coquimbo Region, Chile (32°08′30″ S, 71°30′59″ W) in the spring of November 2024. The plant material was identified by Forestry Engineer Patricio Novoa, and a voucher specimen (HF-1124) was deposited at the Natural Products and Organic Synthesis Laboratory at the Universidad de Playa Ancha, Valparaíso, Chile. The fresh leaves were manually separated and used immediately for extraction. Fresh leaf material (1 kg) was subjected to hydrodistillation using a Clevenger-type apparatus equipped with a Liebig condenser, following the standardized protocol established by our research group [72]. The plant material was distilled with 4 L of deionized water for 4 h at 80 °C. The obtained EO was dried over anhydrous sodium sulfate to remove any residual moisture from the distillation process; subsequently, the drying agent was filtered. The EO yield was estimated using Equation (1), and the resulting 400 mg of oil were maintained in hermetic amber glass vials at 4 °C for further characterization and bioassays.

(1) Yield (%) = (Mass of extracted EO/Mass of dry plant material) × 100

### 4.2. General

The chemicals used were obtained from Sigma-Aldrich (St. Louis, MO, USA) or AK Scientific (Union City, CA, USA) and were used without further purification. These were 4-terpineol (95%), α-bisabolol (90%), carvacrol (98%), 5,5-dithiobis(2-nitrobenzoic acid) (DTNB) (95%), acetylthiocholine iodide (ATChI) (98%), acetylcholinesterase from *Electrophorus electricus* (electric eel), Pluronic^®^ F127 and Span^®^ 80.

### 4.3. Gas Chromatography-Mass Spectrometry (GC/MS) Analysis

The chemical composition of *H. foliosus* EO was analyzed according to the methodology described by Madrid et al. (2026) using a Trace 1300 gas chromatograph coupled to a TSQ8000 Evo triple quadrupole mass spectrometer (Thermo Fisher Scientific, Waltham, MA, USA) [73]. For the analysis, EO samples were diluted in dichloromethane, and 1 µL was injected in splitless mode at an injector temperature of 250 °C. Chromatographic separation was achieved using an RTX-5 ms capillary column (60 m × 0.25 mm i.d., 0.25 μm film thickness). Helium was used as the carrier gas at a constant flow rate of 1.2 mL/min. The oven temperature program was initiated at 40 °C (held for 5 min), then increased to 300 °C at a rate of 5 °C/min, and maintained at the final temperature for 5 min. The mass spectrometer was operated in the electron impact (EI) mode at 70 eV, with the transfer line temperature set at 200 °C. Individual components were identified by comparing their mass spectra with those stored in the NIST20 library and by comparing their linear retention indices, calculated relative to a series of C_8_-C_36_ n-alkanes, with values reported in the literature.

### 4.4. Nanoformulation

Nanoemulsion was prepared following the methodology described by Montenegro et al. (2025), with some modifications [74]. The aqueous (distilled water and Pluronic^®^ F127) and oil (olive oil and Span^®^ 80) phases were pre-heated separately at a temperature of 60 °C. The EO of *H. foliosus* was added to a final concentration of 1000 ppm. The oil phase was added to the aqueous phase and this mixture was stirred in an ultrasonic bath for 10 min at 65 °C. Then, the mixture was subjected to a Vibra-Cell VCX130 Ultrasonic Processor with a ¼ diameter probe (Sonics & Material Inc., Newtown, CT, USA). The vibration amplitude was set at 90% for 6 min, and a pulse cycle of 50 (on cycle)–20 (off cycle) was programmed. An empty nanoemulsion (blank) was prepared following the same procedure described above, replacing the *H. foliosus* EO with deionized water. The nanoemulsion was then stored at room temperature until it was characterized and evaluated.

#### 4.4.1. Determination of Particle Size and Zeta Potential

Prior to measurements, samples were diluted 200-fold with deionized water. The mean particle size and zeta potential of the nanoemulsions were then measured using dynamic light scattering (DLS) and phase analysis light scattering (PALS), respectively, with a Zetasizer Nano ZS system (Malvern Instruments, Malvern, UK) [40].

#### 4.4.2. Stability at Accelerated Storage

The procedure for evaluating the stability of the nanoemulsion was conducted in accordance with the Collaborative International Pesticide Analytical Council (CIPAC) guideline MT 46.6 for accelerated storage procedure (CIPAC https://www.cipac.org/index.php/guidelines, accessed on 16 January 2026) and Abdelmaksoud et al. (2024) with modifications [75]. A total of 10 mL of loaded and empty formulation were placed in separate vials and centrifuged at 5000 rpm for 30 min at room temperature. The vials were then subjected to six cycles of alternating heat and cold at temperatures of 4 °C and 40 °C for 24 h each. For the freezing-temperature evaluation, the formulations were subjected to −18 °C for 48 h. After each stage, whether phase separation occurred was noted.

### 4.5. Insecticidal Assays

#### 4.5.1. Insects and Incubation Condition

Adult *D. melanogaster* specimens were maintained in culture bottles containing an artificial diet composed of brewer’s yeast, sugar, agar, propionic acid and mineral salts. The culture bottles and Petri dishes of the assays were maintained in a VELP 215L cooled incubator (VELP Scientific, Inc., Deer Park, NY, USA) at 24 °C, with a relative humidity of >60% and a photoperiod of 12 h:12 h (L:D).

#### 4.5.2. Determination of the Median Lethal Concentration (LC_50_) in Larvae

The activity of the samples was determined following the methodology described by Muñoz-Nuñez et al. (2025) [76]. Ten first instar larvae were collected from the culture bottles and transferred to a Petri dish containing the same artificial diet with the treatments at concentrations of 10, 25, 50, 100 and 200 µg/mL. The Petri dishes with the different treatments were incubated and larval mortality was recorded at 72 h [76]. All assays were performed in triplicate. The results were compared with those of the solvent control, which contained an untreated artificial diet. The median lethal concentration (LC_50_) was defined as the lethal concentration that achieved 50% mortality of the larvae and was determined by Probit Regression Analysis using Microsoft^®^ Excel^®^ LTSC MSO (Version 2408 Build 16.0.17932.20638).

#### 4.5.3. Inhibitory Activity of AChE Enzyme

The inhibitory activity of the samples on AChE was determined following Ellman’s method with modifications using 96-well plates [76]. Samples were dissolved in DMSO and subsequently diluted in phosphate-buffered saline (PBS, pH 7.4) to prepare a series of twofold dilutions, starting from a maximum concentration of 500 µg/mL. The enzyme solution (0.1 u/mL) was prepared by diluting the commercial stock in PBS. The reaction mixture comprised ATChI as the substrate and DTNB as the chromogenic reagent. In each well, 50 µL of the sample and 50 µL of the enzyme solution were pre-incubated at 37 °C for 30 min. Subsequently, 100 µL of the ATChI/DTNB mixture was added and incubated for an additional 15 min. For the control, the sample was replaced by PBS, while the blank consisted of the sample and substrate without the enzyme. Absorbance was measured at 415 nm using a Cytation 5 Multi-Mode Reader (Agilent BioTek, Santa Clara, CA, USA). The yellow color intensity is proportional to the enzyme activity. Results were expressed as the median inhibitory concentration (IC_50_) calculated via log-linear regression analysis using Microsoft^®^ Excel^®^ LTSC MSO (Version 2408 Build 16.0.17932.20638). All assays were performed in triplicate.

### 4.6. In Silico Study

#### 4.6.1. In Silico Prediction of Physicochemical Properties and Toxicity

Analyses were performed to obtain a preliminary characterization of the physicochemical profile, ADME properties, and potential toxicological behavior of the main compounds evaluated in this study. These predictions were included to support the interpretation of their biological performance, particularly with respect to membrane permeability, aqueous solubility, and their potential suitability for formulation and application. Furthermore, the toxicological predictions were used as an initial approximation of the potential safety profile of these compounds, primarily in relation to human exposure scenarios (e.g., applicators and consumers), rather than as a direct assessment of toxicity to non-target organisms. The toxicological parameters were predicted using the ProTox-III platform (https://tox.charite.de/protox3/index.php?site=compound_search_similarity, accessed on 20 January 2026). In parallel, physicochemical descriptors and parameters associated with absorption, distribution, metabolism, and excretion (ADME) were calculated using the SwissADME web server (https://www.swissadme.ch/, accessed on 23 January 2026). For both platforms, the canonical SMILES strings for each compound were used as input. The SMILES notations were obtained from the PubChem database (https://pubchem.ncbi.nlm.nih.gov/, accessed on 23 January 2026). These computational predictions were considered an exploratory and complementary approach and were not intended to replace specific experimental toxicological or ecotoxicological assays.

#### 4.6.2. Ligand Preparation

The chemical structures of the ligands used in the molecular docking studies were retrieved from the PubChem database in SMILES format. Subsequently, three-dimensional models were generated by editing and converting the files using UCSF Chimera software (version 1.18). During this process, polar hydrogens were added and Gasteiger partial charges were assigned using the Amber ff14SB force field. The molecular geometries were optimized through an energy minimization procedure within the same computational environment, and the resulting structures were finally saved in MOL2 format for use in the docking calculations.

#### 4.6.3. Protein Preparation and Molecular Docking Studies

Molecular docking studies were performed considering the major compounds present in the EO of *H. foliosus*. The crystallographic structure of acetylcholinesterase from *D. melanogaster* [77] (*Dm*-AChE; PDB ID: 6XYS; 2.46 Å resolution), obtained from the Protein Data Bank, was selected as the molecular target. To confirm the location of the active site, the *Dm*-AChE structure was structurally aligned using the MultiSeq module implemented in Visual Molecular Dynamics software (VMD, version 1.9.3), employing acetylcholinesterase from *Torpedo californica* [78] (PDB ID: 1EA5) as a reference. This procedure enabled the identification of conserved regions within the catalytic site and the definition of the area of interest for protein–ligand interaction analyses. Molecular docking was carried out using AutoDock4 for Windows (version 4.2.6), which employs the Lamarckian Genetic Algorithm as the conformational search strategy. The search space was defined using a grid box of 24 × 24 × 24 points, centered at the coordinates X = 28.885, Y = 66.331, and Z = 14.238. During the simulations, the protein structures were treated as rigid, whereas the ligands were allowed full conformational flexibility. The docking parameters were set to perform 50 independent runs per system, with a maximum of 25 million energy evaluations per ligand. All simulations were conducted under physiological pH conditions to approximate a biologically relevant environment.

### 4.7. Statistical

Data from larvicidal assays were subjected to Probit analysis, model fit was verified using the chi-square (χ^2^) test, and values were considered significantly different when the 95% confidence intervals did not overlap. Additionally, an analysis of variance (ANOVA) followed by Tukey’s HSD post hoc test was employed to identify significant differences between the IC_50_ values of the EO, nanoformulations, and pure compounds. Statistical analyses were performed using OriginPro 8.0 software (Version 8.0724). The level of statistical significance was set at *p* < 0.05.

## 5. Conclusions

The present study demonstrates that nanoformulation of *H. foliosus* EO represents a highly effective strategy for the control of *Drosophila* and may be an alternative for the development of new insecticides of natural origin. The results confirm that the nanoencapsulation of the oil significantly enhances the insecticidal activity, achieving a lower LC_50_ compared to the EO in its free state. This effect is supported by the inhibition of the AChE enzyme, where compounds 4-terpineol and α-bisabolol exhibit high binding affinity at the active site, as validated by molecular docking models. Finally, the predicted low human toxicity profile and the exceptional temporal stability of the formulation position it as a viable and sustainable alternative to traditional synthetic insecticides. Beyond these findings, under real-world application conditions, this nanoformulation is expected to provide greater efficacy by ensuring uniform distribution across the surface of crops or even through the immersion of fruits, which could act as a protective barrier against larval development and the transmission of acid rot. Future research should focus on field-scale trials to validate these results, including assessments of environmental effects, efficacy on different fruit matrices, and rigorous safety evaluations for consumers and non-target organisms.

## Figures and Tables

**Table 1:** Main components of *Haplopappus foliosus* EO.

No.	Components	Area (%)	RI ^a^	RL ^b^	Identification
1	α-Terpinene	0.11	1023	1023	RL, MS, Co
2	p-Cymene	2.16	1030	1030	RL, MS, Co
3	Linalool	0.47	1101	1102	RL, MS, Co
4	Fenchol	0.21	1122	1224	RL, MS
5	trans-2-Menthenol	0.80	1129	1130	RL, MS, Co
6	cis-2-Menthenol	0.89	1148	1148	RL, MS
7	endo-Borneol	0.83	1177	1178	RL, MS, Co
8	4-Terpineol	27.27	1188	1188	RL, MS, Co
9	p-Cymen-8-ol	0.65	1193	1194	RL, MS, Co
10	α-Terpineol	2.21	1199	1200	RL, MS, Co
11	cis-Piperitol	0.18	1204	1204	RL, MS
12	trans-Piperitol	0.42	1216	1216	RL, MS
13	Geraniol	0.84	1258	1258	RL, MS, Co
14	γ-Terpinene	1.09	1269	1269	RL, MS
15	trans-Ascaridol glycol	0.18	1282	1282	RL, MS
16	Cuminol	0.69	1294	1294	RL, MS
17	Geranyl formate	0.29	1305	1305	RL, MS
18	Carvacrol	0.58	1309	1310	RL, MS, Co
19	Eugenol	1.12	1322	1322	RL, MS, Co
20	Geranyl acetate	0.95	1383	1384	RL, MS, Co
21	Copaene	0.46	1389	1390	RL, MS
22	Methyleugenol	1.33	1408	1408	RL, MS, Co
23	trans-α-Bergamotene	0.44	1437	1437	RL, MS
24	Caryophyllene	1.11	1448	1448	RL, MS, Co
25	Alloaromadendrene	0.58	1458	1458	RL, MS
26	Selina-5,11-diene	0.13	1462	1462	RL, MS
27	Eudesma-1,4(15),11-triene	2.42	1478	1478	RL, MS
28	γ-Muurolene	0.77	1481	1481	RL, MS
29	Germacrene D	1.18	1491	1491	RL, MS
30	(1S,2S,4S)-trihydroxy-p-menthane	0.53	1497	1497	RL, MS
31	β-Selinene	1.04	1506	1506	RL, MS
32	α-Selinene	2.17	1515	1516	RL, MS
33	α-Farnesene	0.24	1524	1524	RL, MS
34	γ-Cadinene	1.10	1531	1531	RL, MS
35	Cadina-1(10),4-dien-8α-ol	2.32	1538	1538	RL, MS
36	Kessane	0.60	1551	1550	RL, MS
37	Caryophyllene oxide	2.02	1561	1561	RL, MS, Co
38	Globulol	0.71	1582	1582	RL, MS
39	Germacrene-4-ol	0.33	1586	1586	RL, MS
40	Spathulenol	6.70	1600	1600	RL, MS
41	Isoaromadendrene epoxide	2.39	1608	1608	RL, MS
42	Tetradecanal	1.48	1612	1612	RL, MS
43	epi-α-cadinol	0.54	1634	1634	RL, MS
44	Muurola-4,10(14)-dien-1β-ol	3.77	1649	1650	RL, MS
45	γ-Eudesmol	0.88	1654	1654	RL, MS
46	β-Eudesmol	1.66	1657	1658	RL, MS
47	τ-Cadinol	1.45	1661	1662	RL, MS
48	α-Eudesmol	4.95	1676	1676	RL, MS
49	α-Bisabolol	10.40	1699	1699	RL, MS
50	t-Muurolol	0.40	1725	1726	RL, MS
51	Tetradecanoic acid	0.23	1763	1762	RL, MS
52	10-epi-γ-eudesmol	0.22	1773	1773	RL, MS
53	Nootkatone	0.35	1814	1814	RL, MS, Co
54	Hexadecanol	0.27	2120	2218	RL, MS
55	Tetracosane	0.79	2294	2294	RL, MS
56	Pentacosane	0.15	2494	2484	RL, MS
	Total identified	98.05			
	Hydrocarbon monoterpenes	3.36			
	Oxygenated monoterpenes	36.17			
	Monoterpene esters	1.24			
	Hydrocarbon sesquiterpenes	11.64			
	Oxygenated sesquiterpenes	39.69			
	Phenols	0.58			
	Phenylpropanoids	2.45			
	Hydrocarbon alkane	0.94			
	Fatty alcohol	0.27			
	Fatty aldehyde	1.48			
	Fatty acid	0.23			

Area: surface area of GC peak; ^a^ RI: experimental retention index for non-polar column; ^b^ RL: bibliographic retention index for non-polar column; MS: mass spectra; Co: co-elution with standard compounds available in our laboratory.

**Table 2:** Characterization after 7 days of storage of empty nanoemulsion (blank) and the *Haplopappus foliosus* essential oil nanoformulation.

Formulation	Ph	Particle Size (nm)	PDI	ZP (mV)
EO-loaded nanoemulsion	6.92	2.10 ± 0.13	0.200	−3.38 ± 0.2
Unloaded nanoemulsion	6.89	2.80 ± 0.14	0.194	−3.44 ± 0.1

PDI: polydispersity index; ZP: zeta potential.

**Table 3:** Evaluation of the stability of nanoemulsions (loaded and unloaded) against temperature and mechanical variables after 90 days of storage.

Formulation	Particle Size (nm)	PDI	Centrifugation	Temperature Cycle	Freezing
EO-loaded nanoemulsion	1.98 ± 0.00	0.200	NS	NS	NS
Unloaded nanoemulsion	242.5 ± 0.00	0.289	NS	NS	NS

PDI: polydispersity index; NS = no phase separation.

**Table 4:** Mortality of *Drosophila melanogaster* first instar larvae treated with *Haplopappus foliosus* EO, its nanoformulation and phytochemicals.

Sample	LC_50_ (µg/mL)	95% CI (µg/mL)		Slope ± SE	χ^2^
		Lower	Upper		
*H. foliosus* EO	120.26 ^d^	107.17	134.92	1.67 ± 0.15	0.58
EO-loaded nanoemulsion	54.57 ^b^	52.40	56.83	1.18 ± 0.10	0.20
4-terpineol	76.85 ^c^	70.21	84.11	1.61 ± 0.14	1.25
α-bisabolol	82.36 ^c^	78.72	86.16	1.93 ± 0.13	3.10
Empty nanoemulsion	>200 ^e^	-	-	-	-
C+	119.91 ^d^	117.18	122.71	1.88 ± 0.03	1.99
M+	0.11 ^a^	0.10	0.12	9.75 ± 0.17	0.32

CI: confidence limits; C+: positive control carvacrol; M+: positive control malathion; χ^2^: chi square test; -: not applicable. Different letters in the superscript in the same column represent significant differences.

**Table 5:** Inhibitory activity of *Haplopappus foliosus* EO, its nanoemulsion, and main constituents on AChE.

Sample	IC_50_ ± SE (µg/mL)
*H. foliosus* EO	115.38 + 2.2 ^d^
EO-loaded nanoemulsion	166.85 + 1.6 ^e^
4-terpineol	77.13 + 1.3 ^b^
α-bisabolol	89.76 + 0.5 ^c^
Empty nanoemulsion	>500 ^f^
C+	55.93 + 0.6 ^a^

C+: positive control carvacrol. Results are expressed as mean ± standard error. Different letters in the superscript in the same column represent significant differences.

**Table 6:** Predicted physicochemical and ADME-related properties of the evaluated compounds calculated using SwissADME.

Compound	MW (g/mol)	LogP	TPSA (Å^2^)	Solubility in Water	HBD	HBA	RB	GI	Pains
4-terpineol	154.25	2.6	20.23	Poorly soluble	1	1	1	High	0
α-bisabolol	222.37	3.8	20.23	Poorly soluble	1	1	4	High	0
Carvacrol	150.22	2.8	20.23	Poorly soluble	1	1	1	High	0

MW: molecular weight; LogP: lipophilicity; TPSA topological polar surface area; HBD: number of hydrogen bond donors; HBA number of hydrogen bond acceptor; RB: number of rotatable bonds; GI: gastrointestinal absorption. LogP values correspond to the average of iLOGP, XLOGP3, WLOGP, MLOGP, and SILICOS-IT models. Solubility and GI absorption are qualitative classifications provided by SwissADME. PAINS indicates the number of structural alerts.

**Table 7:** Toxicological data of major compounds and positive control obtained from the ProTox-III database.

Compound	OT	TC	HT	NT	NPHT	RT	CT	BBB
4-terpineol	1016	4	Inactive	Inactive	Inactive	Inactive	Inactive	Active
α-bisabolol	2830	5	Inactive	Inactive	Inactive	Inactive	Inactive	Active
Carvacrol	810	4	Inactive	Active	Inactive	Active	Inactive	Active

OT: oral toxicity (mg/kg); TC: toxicity class according to Globally Harmonized System (GHS); HT: hepatotoxicity; NT: neurotoxicity; NPHT: nephrotoxicity; RT: respiratory toxicity; CT: cardiotoxicity; BBB: blood–brain barrier.

**Table 8:** Binding energies of the major compounds present in the *Haplopappus foliosus* EO and positive control carvacrol.

Compound	Binding Energies (kcal/mol) ^a^
4-terpineol	−8.6
α-bisabolol	−8.0
Carvacrol	−5.7

^a^ Binding energies correspond to the best docking pose.

## Data Availability

The original contributions presented in this study are included in the article. Further inquiries can be directed to the corresponding author.
